# Supplementary material for: Determination of server location in emergency care systems: an index proposal using data envelopment analysis and the hypercube queuing model
Source: PeerJ Comput Sci. 2023 Nov 3;9:e1637. doi: 10.7717/peerj-cs.1637 (PMC10703110; doi:10.7717/peerj-cs.1637)
Supplement: Supplemental Information 1 [file peerj-cs-09-1637-s001.docx]

**Appendix**

This appendix presents the generalization of the state-to-state transitions of the hypercube model (Beojone et al., 2020). Equation A.1 shows the major equation of this model:

|  | $P_{r}\left[ L\left( r \right)\sum_{j} \sum_{l} I\left( R\left( r,jl \right)\neq\emptyset\right)\lambda_{jl}+\left( 1-L\left( r \right) \right)\lambda+\sum_{t} S\left( r,k \right)\mu_{t} \right]=\sum_{q,t:D\left( q,r,t \right)=1} P_{q}S\left( q,t \right)\mu_{t}+\sum_{q,l:Q\left( q,r,l \right)=1} P_{q}\sum_{t} S\left( q,t \right)\mu_{t}+\sum_{q,t:D\left( r,q,t \right)=1} P_{q}\sum_{jl\in A(q,t)} \lambda_{jl}+P_{q}\sum_{q,l:Q\left( r,q,l \right)=1} \lambda_{jl}$ | (A.1) |
| --- | --- | --- |

In Equation A.1, q and r are the states belonging to the set of states M. P_r_ and P_q_ are the probabilities of the steady states r and q, respectively, while t and jl are defined as the groups of servers and subatoms of the system, respectively. I($*$) is the indicator of a function (1, if $I(*)$ is true, otherwise 0). L(r) indicates whether state r is likely to miss a call. R (r, jl) represents the set of servers that are still available to serve subatom jl in the state r. $S\left( r,t \right)$ shows the number of servers in the group t occupied in the state r. The arrival rate from subatom jl is λ_jl_, while λ is the arrival rate for all subatoms. The service rate of each group t is µ_t._ The service rate is obtained considering the server's journey time and on-site service time. In addition, as server locations vary from the original scenario, there is a need to calibrate the service times *μ*^-1^ (Larson & Odoni, 2007). The process is similar to the calculation of the initial response times, using the average service time and the journey time of the servers in the original scenario. Here, the service time is the same, but the journey time considered is that of the last solution of the hypercube model for the given configuration. The process is repeated until the changes in the service times of the servers are below a tolerance of 0.1. $A\left( q,t \right)$ is the set of subatoms in which the server group t is preferable in the state q (based on the dispatch system preference list). $D(q,r,t)$ is another function with definition based on Equation A.2:

|  | $D\left( q,r,t \right)=\left\{ \begin{aligned} 1, & if H\left( q,r \right)=1, S\left( q,t \right)=S\left( r,t \right)+1 \\ 0, &otherwise \end{aligned} \right.$ | (A.2) |
| --- | --- | --- |

Where *H (q, r)* is the minimum number of transitions between states *q* and *r*. $Q\left( q,r,l \right)$ is another function defined in Equation A.3 and is related to the transitions between the queue states:

|  | $D\left( q,r,t \right)==\left\{ \begin{aligned} 1, & if H\left( q,r \right)=1, S\left( q,k \right)=S\left( r,t \right) \forall k, W\left( q,r,l \right)=1 \\ 0, &otherwise \end{aligned} \right.$ | (A.3) |
| --- | --- | --- |

In this equation, $W\left( q,r,l \right)$ represents the function that indicates the number of classes $l$ under service, in which state q has more states than in r.
